# Face Masks Do Not Alter Gaze Cueing of Attention: Evidence From the COVID-19 Pandemic

**DOI:** 10.1177/20416695211058480

**Published:** 2021-11-30

**Authors:** Mario Dalmaso, Xinyuan Zhang, Giovanni Galfano, Luigi Castelli

**Affiliations:** Department of Developmental and Social Psychology, 9308 University of Padova, Padova, Italy; Department of Developmental and Social Psychology, University of Padova, Padova, Italy;; Department of Psychology and Center for Brain and Cognitive Sciences, School of Education, 47875 Guangzhou University, Guangzhou, China; Department of Developmental and Social Psychology, University of Padova, Padova, Italy

**Keywords:** face mask, social attention, gaze cueing, COVID-19 emergency

## Abstract

Interacting with others wearing a face mask has become a regular worldwide practice since the beginning of the COVID-19 pandemic. However, the impact of face masks on cognitive mechanisms supporting social interaction is still largely unexplored. In the present work, we focused on gaze cueing of attention, a phenomenon tapping the essential ability which allows individuals to orient their attentional resources in response to eye gaze signals coming from others. Participants from both a European (i.e., Italy; Experiment 1) and an Asian (i.e., China; Experiment 2) country were involved, namely two countries in which the daily use of face masks before COVID-19 pandemic was either extremely uncommon or frequently adopted, respectively. Both samples completed a task in which a peripheral target had to be discriminated while a task irrelevant averted gaze face, wearing a mask or not, acted as a central cueing stimulus. Overall, a reliable and comparable gaze cueing emerged in both experiments, independent of the mask condition. These findings suggest that gaze cueing of attention is preserved even when the person perceived is wearing a face mask.

## Introduction

From the COVID-19 outbreak onwards, face mask has been introduced by governments worldwide as a protective gear to stop the spread of the virus (e.g., Cheng et al., 2020; Eikenberry et al., 2020). From a practical perspective, wearing a face mask is aimed at preventing salivary particles from being transmitted by air from one individual to another, thus reducing the possibility of a potential contagion (e.g., Liang et al., 2020; van der Sande et al., 2008). However, despite the fundamental role of face masks in promoting public health, it is undoubted that they can also interfere with interpersonal relations to a large extent. In particular, by occluding a relatively large part of the face, the presence of a face mask prevents an observer from properly perceiving and processing a number of information coming from others.

From a psychological perspective, faces have a profound impact on a variety of cognitive mechanisms supporting social interaction. For instance, from the unchangeable features of others’ face we can acquire information concerning their age, gender, and, more in general, identity (Haxby et al., 2000). Similarly, facial expressions can be used to infer others’ feelings and mental states (Adams et al., 2006). Moreover, eye gaze direction of others allows us to infer where they are attending to, thus allowing us to orient our own attention towards the same spatial location (for reviews, see Capozzi & Ristic, 2018; Dalmaso et al., 2020b; Frischen et al., 2007). For these reasons, it is highly likely that wearing a face mask during social interactions could reasonably interfere with specific mechanisms involved in face processing. Supporting evidence to this notion comes from recent studies showing reduction in both identity recognition (e.g., Freud et al., 2020; Noyes et al., 2021; for other related evidence see also Giovanelli et al., 2021) and emotion recognition (e.g., Carbon, 2020; Marini et al., 2021; Nestor et al., 2020; Parada-Fernández et al., 2022) of faces wearing a face mask. However – to the best of our knowledge – so far, no studies investigated the potential impact of face masks on social attention. The present work aims at filling this gap.

Social attention has been widely studied by adopting the so-called gaze cueing task (e.g., Driver et al., 1999; Friesen & Kingstone, 1998), which typically consists in presenting, at the beginning of each trial, a task irrelevant central face with the gaze averted either leftwards or rightwards (i.e., the cue). After a variable temporal duration (i.e., Stimulus Onset Asynchrony, SOA), a peripheral target appears, and individuals are required to provide a manual response. The results generally show that response latencies are smaller when the target appears on a spatial location gazed at by the central face (i.e., a spatially congruent trial), rather than elsewhere (i.e., a spatially incongruent trial). This pattern, in turn, is thought to reflect the tendency of averted gaze faces to elicit attentional shifts towards the same location (i.e., the gaze cueing effect). Here, a gaze cueing task was adopted in which the face providing the gaze cue belonged to an individual who could either wear a face mask or not, in order to reveal the potential impact of this protective gear on gaze-mediated orienting of attention. In this regard, it is worth noting that some previous studies (e.g., Akiyama et al., 2008; Hayward & Ristic, 2015; Kingstone et al., 2000; Slessor et al., 2016) reported a reliable gaze cueing effect even when participants were presented with just two eye gaze stimuli in isolation, rather than embedded within a whole face stimulus – an approach that closely resembles the perceptual condition associated with a face wearing a mask, for which the eyes are the only visible part. Furthermore, Quadflieg et al. (2004) reported a reliable gaze cueing effect also when eye gaze stimuli were embedded in schematic animal faces (e.g., a tiger or a monkey) or in inanimate schematic non facial stimuli (i.e., an apple or a glove). Importantly, this effect was similar in magnitude to the gaze cueing effect emerging from a schematic human face. Taken together, these results seem to suggest that gaze cueing of attention can be elicited without necessarily presenting participants with a whole human face and therefore a reliable gaze cueing effect should reasonably emerge even for faces wearing a face mask. Nevertheless, it is also important to note that all the above studies either used artificial, schematic stimuli (i.e., drawings) or stimuli extracted from pictures of real faces (i.e., the eye region) that, however, were presented in highly impoverished contexts that do not resemble real-life situations. In addition, it is worth remarking that, in recent years, a great bulk of studies clearly showed that both individual characteristics and contextual factors can deeply shape social attention when more ecological stimuli are used instead (Dalmaso et al., 2020b). Of particular relevance for the present work, individual levels of fearfulness (Tipples, 2006), threatening contexts (Ohlsen et al., 2013) or faces associated with or communicating a potential threat (Chen & Zhao, 2015; Kuhn & Tipples, 2011; Kuhn et al., 2015) are all known to potentiate the gaze cueing effect, likely reflecting an increased monitoring of the surrounding environment possibly aimed at implementing defensive behaviours. For this reason, on the one hand, in a context characterised by an aggressive and dangerous airborne disease – such as the COVID-19 – an enhanced gaze cueing could be expected in response to individuals not wearing a face mask, since these could be perceived as a possible source of contagion. Importantly, the fear of contagion can be triggered also by using pictorial stimuli presented on a computer monitor (e.g., Schaller et al., 2010). However, on the other hand, a recent study (Olivera-La Rosa et al., 2020) has also shown that individuals wearing a face mask can be perceived as more likely to be sick – but also more trustworthy, a characteristic that can boost gaze cueing (see, e.g., Süßenbach & Schönbrodt, 2014) – and therefore it cannot be excluded that an enhanced gaze cueing could instead emerge for faces wearing a face mask. These two competing hypotheses were tested in the present study. Self-reported measures of objective habits and subjective viewpoints towards face mask usage and the perception of infection risk were also collected, in order to explore any potential link between individual attitudes and gaze cueing. In addition, we also assessed whether the impact of face mask on gaze cueing may vary in different cultural backgrounds. More specifically, the gaze cueing task we devised was delivered to both a sample of European individuals (i.e., Italians) living in a European country (i.e., Italy) and to a sample of Asian individuals (i.e., Chinese) living in an Asian country (i.e., China). Indeed, while in Western countries, before the COVID-19 emergency, the use of face masks in everyday life was extremely uncommon, this cannot be said for several Eastern countries – and especially for China – in which, after the SARS outbreak of 2003, individuals are more generally inclined to wear a mask for a variety of reasons related to health, such as reducing the spread of common cold in public places (e.g., Meng et al., 2021; Nie et al., 2015; see also Sin, 2016). On this basis, we also explored whether the possible difference in gaze cueing magnitude elicited by faces wearing a mask or not – if any – could be further shaped by cultural context.

To sum up, in the present study, we conducted two experiments based on a gaze cueing task in which averted gaze faces, either wearing a face mask or not, were employed as cueing stimuli. The gaze cueing task was delivered to a sample of Italian individuals living in Italy (Experiment 1) and to a sample of Chinese individuals living in China (Experiment 2). Because ethnic membership can deeply shape gaze cueing (e.g., Dalmaso et al., 2015; Weisbuch et al., 2017; Zhang et al., 2021), in both Experiments participants were presented with faces belonging to their own ethnic group. Self-reported measures assessing COVID-related habits and perceptions were also collected. In so doing, we explored the potential impact of face mask on gaze cueing of attention under different perspectives.

## Experiment 1: Face Mask and Gaze Cueing in a European Country

### Materials and Methods

#### Participants

Data were collected from October 23, 2020, until November 7, 2020, during the second wave of the pandemic in Italy, which took place in the fall period. In particular, during the time window in which data were collected, the new daily cases of COVID-19 in Italy increased from 19.143 to 39.809, according to the COVID-19 data repository by Dong et al. (2020).

The sample was composed of Italian students at the University of Padova, Italy, who took part on a voluntary basis. Based on previous studies in which a whole face vs. only eyes were visible (e.g., Hayward & Ristic, 2015; Quadflieg et al., 2004; Slessor et al., 2016), we aimed at collecting data from about 40 participants. Data collection was stopped, for convenience, at *N* = 46 (*Mean age* = 21.35 years, *SD* = 6.12, 11 males). Before the experiment, participants were asked to read an informed consent, which was provided by pressing a specific keyboard key. The project was conducted in line with the Declaration of Helsinki, and it was approved by the Ethics Committee for Psychological Research at the University of Padova.

#### Stimuli and Procedure

Facial stimuli consisted of high resolution faces (300 px width × 450 px height), depicting two White males and two White females with neutral expression, extracted from the MR2 database (Strohminger et al., 2016; see also Strachan et al., 2017). For each identity, there was the original stimulus with direct gaze and two new stimuli of the same face with gaze averted both leftwards and rightwards. Moreover, we also created, for each of these three stimuli, three additional new faces wearing a face mask. Hence, for each facial identity, there were six different versions in total. All stimuli were presented in full colour against a white background. The experiment was created with PsychoPy software and administered online by using Pavlovia, which allow to collect precise and reliable behavioural data across different operating systems and web browsers (see also Bridges et al., 2020). The experiment could only be completed through the use of a computer. At the beginning of the experiment, the software randomly selected which individuals (one male and one female), among the four different identities, would be systematically presented to a given participant as either wearing a mask or not.

**Figure 1.**
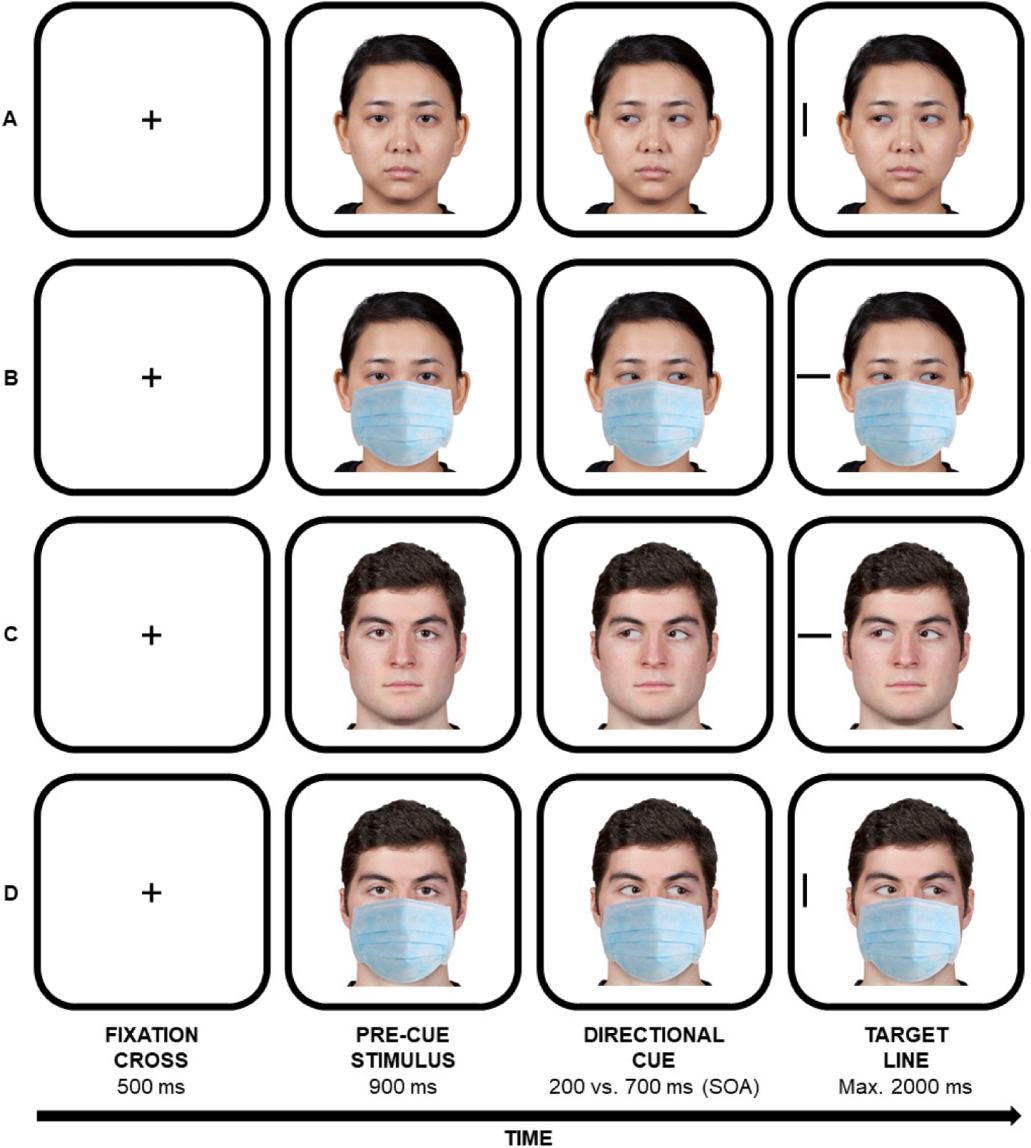
Examples of faces (not drawn to scale) and trials composing the gaze cueing task. A female Asian individual (panels A and B) and a male white individual (panels C and D) are depicted, both wearing a face mask or not. Congruent trials are those in which the face looks towards the spatial location in which the target line appears (panels A and C), whereas incongruent trials are those in which the face looks towards the opposite spatial location with respect to the target line (panels B and D).

In the gaze cueing task (please see Figure 1), each trial started with a black fixation cross (0.1 normalized units) presented at the centre of the screen for 500 ms. Then, a central face with the gaze directed to the participant appeared for 900 ms. After either 200 ms or 700 ms (SOA), a black target line (40 px width × 12 px height) appeared either leftwards or rightwards (±0.8 normalized units with respect to the centre of the screen) until a manual response was provided, or until timeout (2000 ms). Two different SOAs were employed in order to explore the time-course of any possible modulations of gaze cueing as a function of the mask condition. The target line could be oriented either horizontally or vertically. Participants were asked to look at the centre of the screen for the whole duration of the trial and to discriminate, as fast and accurate as possible, the orientation of the target line by using two response keys (“f” or “k” key) on the keyboard. The response mapping between line orientation and keys was randomly selected across participants. Participants were also instructed to ignore gaze direction of the facial stimulus since it was not informative about the location of the upcoming target (i.e., the target had the same probability to occur at either the gazed or the non-gazed at location). Trials associated with either a wrong or a missed response were signalled through a visual feedback (i.e., the words ‘NO’ or ‘TOO SLOW’, respectively; Arial font, 0.1 normalized units), provided at the centre of the screen for 500 ms, and they were not replaced by additional trials.

There was a practice block, composed of 10 trials, followed by two experimental blocks, each composed of 128 trials. In so doing, each participant completed 256 experimental trials in total. Within each of the two experimental blocks, each experimental condition was selected in a random order and presented for an equal number of times. Therefore, trials presenting faces with either a face mask or not were intermixed within each block. The gaze cueing task was then followed by a short questionnaire aimed at collecting self-reported measures assessing COVID-related habits and attitudes (please see Appendix A). Before both the gaze cueing experiment and the questionnaire, a specific text screen appeared containing the instructions to successfully complete the requested task.

### Results

Data of all participants who completed the experiment were analysed. Partial data provided by participants who aborted the task before its ending were not stored by the Pavlovia platform. Data analyses were performed through JAMOVI software (https://www.jamovi.org/).

#### Gaze Cueing Task

Missing responses (0.34% of trials) were rare and therefore not further analysed. Wrong responses (4.70% of trials) were analysed separately. Outliers, namely correct trials with a latency smaller than 150 ms or greater than 1500 ms (0.31% of trials), were discarded from RT analyses. Data were analysed both using a frequentist approach and a Bayesian framework, in order to establish the best model fitting the available data.

**Figure 2.**
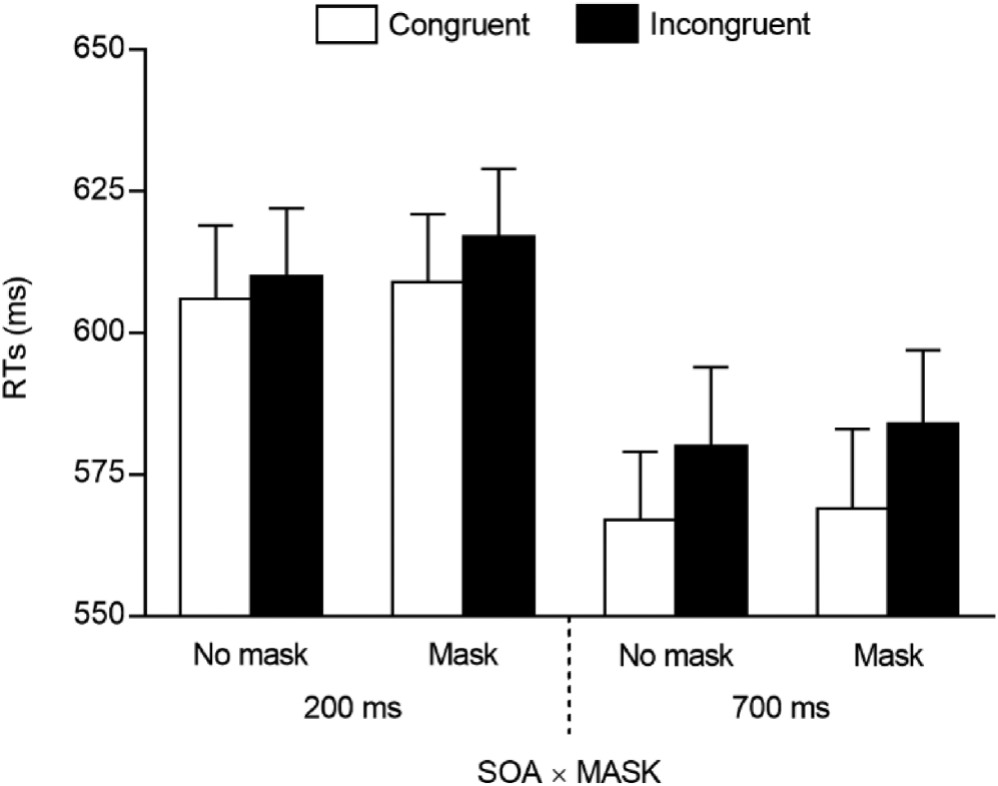
Mean RTs observed in Experiment 1 (European participants) as a function of spatial congruency, SOA, and mask. Error bars are SEM.

RTs for correct trials were analysed through a repeated measures ANOVA, with spatial congruency (2: congruent vs. incongruent), SOA (2: 200 vs. 700 ms), and mask (2: mask vs. no mask) as within-participants factors. The main effect of spatial congruency was significant, *F*(1, 45) = 12.378, *p* < .001, *η^2^_p_* = .216, due to smaller RTs on congruent (*M* = 588 ms, *SE* = 12.03) than on incongruent (*M* = 598 ms, *SE* = 12.53) trials, as well as the main effect of SOA, *F*(1, 45) = 125.698, *p* < .001, *η^2^_p_* = .736, due to smaller RTs at the longer (*M* = 575 ms, *SE* = 12.71) than at the shorter (*M* = 610 ms, *SE* = 11.89) SOA, consistent with a foreperiod effect. No other results were significant (*F*s < 2.334, *p*s > .134), including the two theoretically relevant spatial congruency × mask, and spatial congruency × SOA × mask interactions (*F*s < 1, *p*s > .760; see also Figure 2). The repeated measures Bayesian ANOVA, performed through the default JAMOVI priors (i.e., r scale fixed effect: .5; r scale fixed effect: 1; r scale covariates: .354), included the same factors as those reported for the frequentist approach. Model comparison included all possible models (i.e., from the null model to the most saturated one), which were compared against the best one. This ANOVA indicated that the best model fitting the data included only the main effects of spatial congruency and SOA. According to Lee and Wagenmakers (2013), this model received extreme evidence (*BF_10_* > 100) for being preferable over the null model, and strong evidence (*BF_10_* = 19.835) for being preferable over the first best model including the theoretically relevant interaction between spatial congruency and mask (i.e., a model with the main effects of spatial congruency, SOA, and mask, and the spatial congruency × mask interaction).

Wrong responses were analysed through an identical ANOVA as that used for RT analyses. The main effect of SOA was significant, *F*(1, 45) = 5.041, *p* = .030, *η^2^_p_* = .101, with fewer errors at the longer (*M* = 4.21%, *SE* = .43) than at the shorter (*M* = 5.2%, *SE* = .48) SOA. No other results were significant (*F*s < 2.512, *p*s > .120). The Bayesian ANOVA, performed identically as that used for RTs analyses, confirmed that the model including only the main effect of SOA was the best one fitting the data. This model received anecdotal evidence (*BF_10_* = 2.228) for being preferable over the null model, and extreme evidence (*BF_10_* > 100) for being preferable over the first best model including the theoretically relevant interaction between spatial congruency and mask (i.e., a model with the main effects of spatial congruency, SOA, and mask, and the spatial congruency × mask interaction).

#### Relationship Between Gaze Cueing and Self-Reported Measures

The mean scores observed for each item of the questionnaire (see Appendix A) are reported, separately for each experiment, in Table 1. In order to assess the presence of a potential relationship between gaze cueing and the self-reported measures, a new index expressing the magnitude of the gaze cueing effect (i.e., mean RTs on incongruent trials – mean RTs on congruent trials) was calculated separately for each mask condition. This index was then correlated with the mean score obtained for both objective and subjective measures. Both classical and Bayesian correlations (Pearson's coefficient) were performed. In particular, since responses to the three items concerning objective measures (Section 1 of the questionnaire) were not intercorrelated (Cronbach's *α* = .20), they were kept separated; responses to the items concerning the subjective measures (Section 2 of the questionnaire) were intercorrelated (Cronbach's *α* = .74), and therefore they were averaged, thus obtaining a single score. Nevertheless, no significant correlations emerged for both the objective (*p*s > .087, *BF_10_*_s_ < 1) and the subjective (*p*s > .224, *BF_10_*_s_ < 1) measures. For completeness, correlations were also performed with an overall index of gaze cueing (calculated by collapsing the data for both the mask and the no mask condition) and non-significant results emerged for both the objective (*p*s > .187, *BF_10_*_s_ < 1) and the subjective (*p* = .481, *BF_10_* < 1) measures.

**Table 1.** Mean Values (and SEM, in parentheses) Obtained From the Five-Point Response Scales, for All the Questionnaire Items (see also Appendix A).

	Section 1			Section 2		
	A1	B1	C1	A2	B2	C2
Experiment 1	4.02 (.17)	4.50 (.09)	4.30 (.11)	4.63 (.08)	3.28 (.15)	3.22 (.18)
Experiment 2	3.54 (.20)	2.96 (.15)	3.39 (.17)	4.15 (.14)	2.54 (.15)	2.52 (.18)

## Experiment 2: Face Mask and Gaze Cueing in an Asian Country

### Materials and Methods

#### Participants

Data were collected from November 11, 2020, until November 13, 2020. During the time window in which data were collected, the new daily cases of COVID-19 in China were extremely rare and changed from 15 to 18, according to the COVID-19 data repository (Dong et al., 2020).

We aimed to collect a similar number of participants as in Experiment 1. The sample was composed of 46 Asian students at the University of Guangzhou, China, who took part on a voluntary basis. Data collection was stopped at N = 46 (*Mean age* = 19.13 years, *SD* = .86, 14 males). The informed consent was provided by participants as in Experiment 1.

#### Stimuli and Procedure

Everything was identical to Experiment 1, with only one exception: Faces belonged to Asian individuals. Importantly, since the MR2 database (Strohminger et al., 2016) provides rating scores for some social dimensions that can be also involved in shaping gaze cueing of attention (i.e., age, masculinity, mood and trust; see Dalmaso et al., 2020b), Asian faces were therefore selected in order to match White faces along all these four dimensions (all *p*s > .27).

### Results

Data of all participants who completed the experiment were analysed. Partial data provided by participants who aborted the task before its ending were not stored by the Pavlovia platform. Data were analysed as in Experiment 1.

#### Gaze Cueing Task

Missing responses (1.51% of trials) were rare and therefore not further analysed. Wrong responses (7.86% of trials) were analysed separately. Outliers (0.76% of trials) were discarded from RT analyses.

**Figure 3.**
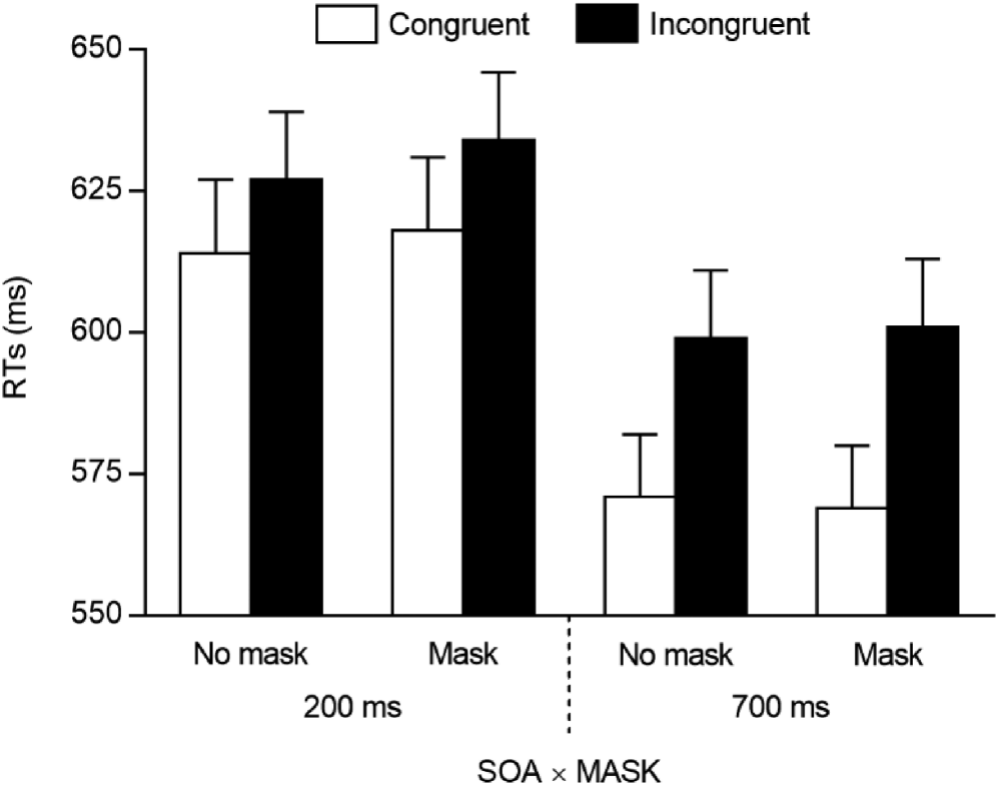
Mean RTs observed in Experiment 2 (Asian participants) as a function of spatial congruency, SOA, and mask. Error bars are SEM.

RTs of correct trials were analysed through a repeated measures ANOVA, with congruency (2: congruent vs. incongruent), SOA (2: 200 vs. 700 ms), and mask (2: mask vs. no mask) as within-participants factors. The main effect of spatial congruency was significant, *F*(1, 45) = 55.335, *p* < .001, *η^2^_p_* = .552, due to smaller RTs on congruent (*M* = 593 ms, *SE* = 11.26) than on incongruent (*M* = 615 ms, *SE* = 11.27) trials, as well as the main effect of SOA, *F*(1, 45) = 83.247, *p* < .001, *η^2^_p_* = .649, due to smaller RTs at the longer (*M* = 585 ms, *SE* = 10.65) than at the shorter (*M* = 623 ms, *SE* = 12.03) SOA. The spatial congruency × SOA interaction was also significant, *F*(1, 45) = 6.047, *p* = .018, *η^2^_p_* = .118, and it was further explored, for each SOA, through two tailed paired t-tests between congruent and incongruent trials. These indicated that the gaze cueing effect emerged both at the 200-ms SOA, *t*(45) = 3.342, *p* = .002, *d* = .493, and at the 700-ms SOA, *t*(45) = 6.610, *p* < .001, *d* = .975, but it was greater in the latter case (14 vs. 30 ms). No other results were significant (*F*s < 1, *p*s > .389), including the two theoretically relevant spatial congruency × mask, and spatial congruency × SOA × mask interactions (*F*s < 1, *p*s > .643; see also Figure 3). The Bayesian ANOVA, including the same factors as those reported for the frequentist approach, was also conducted. This indicated that the best model fitting the data included the main effects of congruency and SOA, and their interaction. This model received extreme evidence (*BF_10_* > 100) for being preferable over the null model, and very strong evidence (*BF_10_* = 40.087) for being preferable over the first best model including the theoretically relevant interaction between spatial congruency and mask (i.e., a model with the main effects of spatial congruency, SOA, and mask, and the spatial congruency × SOA and spatial congruency × mask interactions).

Wrong responses were analysed through an identical ANOVA as that used for RTs analyses. The main effect of SOA was significant, *F*(1, 45) = 16.955, *p* < .001, *η^2^_p_* = .274, with fewer errors at the longer (*M* = 6.44%, *SE* = .76) than at the shorter (*M* = 9.29%, *SE* = 1.14) SOA. No other results were significant (*F*s < 1, *p*s > .520). The Bayesian ANOVA confirmed that the model including only the main effect of SOA was the best one fitting the data. This model received extreme evidence (*BF_10_* > 100) for being preferable over the null model, and extreme evidence (*BF_10_* > 100) for being preferable over the first best model including the theoretically relevant interaction between spatial congruency and mask (i.e., a model with the main effects of spatial congruency, SOA, and mask, and the spatial congruency × mask interaction).

#### Relationship Between Gaze Cueing and Self-Reported Measures

Mean scores collected through the questionnaire are reported in Table 1. As in Experiment 1, correlational analyses between the index of gaze cueing magnitude for faces with or without mask and the scores for the three objective measures (which were only weakly intercorrelated, Cronbach's *α* = .44) and the average index for subjective measures (Cronbach's *α* = .76) did not show any significant result (*p*s > .106, *BF_10_*_s_ < 1). This holds true also for the overall index of gaze cueing (*p*s > .117, *BF_10_*_s_ < 1).

#### Comparison Between Experiment 1 (European Participants) and 2 (Asian Participants)

In order to explore whether the gaze cueing effect was different between the two samples, we conducted a mixed design ANOVA, with the same within-participants factors used in previous analyses (i.e., spatial congruency, SOA, and mask), and with experiment (2: Experiment 1 vs. Experiment 2) as an additional between-participants factor. Both the main effects of spatial congruency and SOA were significant (*F*s > 61.508, *p*s < .001), as well as the spatial congruency × SOA interaction, *F*(1, 90) = 7.758, *p* = .007, *η^2^_p_* = .079, reflecting that the gaze cueing effect, albeit significant at both SOAs (*t*s > 3.366, *p*s < .001), was greater at the longer one. Moreover, the main effect of experiment was not significant, *F*(1, 90) = .461, *p* = .499, *η^2^_p_* = .005, but the experiment × congruency interaction was significant, *F*(1, 90) = 9.301, *p* = .003, *η^2^_p_* = .094, reflecting that the gaze cueing effect, albeit significant in both samples (*t*s > 3.518, *p*s < .001), was greater among Asian than European participants (i.e., 22 vs. 10 ms). More importantly, experiment was not involved in any interaction including both spatial congruency and mask (*F*s < .027, *p*s > .870), thus indicating a similar gaze cueing effect in response to faces irrespective of whether wearing a mask or not, in both samples. The Bayesian ANOVA, including the same factors as those reported for the frequentist approach, indicated that the best model fitting the data included the main effects of spatial congruency, SOA, and experiment, as well as the spatial congruency × SOA and spatial congruency × experiment interactions. This model received extreme evidence (*BF_10_* > 100) for being preferable over the null model, and extreme evidence (*BF_10_* > 100) for being preferable over the first best model including the theoretically relevant interaction between spatial congruency and mask (i.e., a model with the main effects of spatial congruency, SOA, and mask, experiment, as well as the spatial congruency × SOA, spatial congruency × mask, spatial congruency × experiment, mask × experiment, and spatial congruency × mask × experiment interactions).

As a final note, the effect size of the difference between the magnitude of gaze cueing in the mask and the no mask condition (Cohen's *d* = 0.058) suggests that regardless of the statistical significance, the difference, if any, would reflect a practically-irrelevant effect.

## General Discussion

The aim of this work was to assess the possible impact of face mask on a specific aspect related to social attention, namely gaze-driven orienting. In two experiments, we employed a gaze cueing task in which a task irrelevant face, which could either wear a face mask or not, looked either leftwards or rightwards while the participant provided a manual response to a peripheral target. This task was delivered to both a sample of European and Asian individuals, for whom the everyday use of face mask before COVID-19 emergency was either extremely uncommon or far more diffused (Meng et al., 2021; Nie et al., 2015; see also Sin, 2016), respectively. Overall, a reliable gaze cueing effect emerged and, interestingly, its magnitude was not modulated by mask condition, and it was not linked to self-reported measures related to both personal habits and attitudes towards face mask usage and possible infection risk. Moreover, these patterns of results were virtually identical in both the Italian and the Chinese sample, thus suggesting that participants’ cultural background was not involved in shaping the social orienting response investigated here. Importantly, these results found support not only within a frequentist approach, but also within a Bayesian framework, which confirmed that the evidence indicating a possible impact of face mask on gaze cueing of attention was basically null.

Although it has been shown that the gaze cueing effect can be obtained also by presenting eye gaze stimuli in isolation (e.g., Akiyama et al., 2008; Hayward & Ristic, 2015), it is worth remarking that previous studies invariably used either artificial or heavily impoverished stimuli. In addition, the presence of gaze cueing in those studies does not rule out the possibility that gaze cueing might be either magnified or decreased as compared to a condition in which full access to facial information is enabled. Importantly, in our study, stimuli with and without the mask were presented intermixed within the same block of trials, rather than in separate blocks. This manipulation was aimed to maximize the perceptual saliency of the distinctive features of the two sets of stimuli (i.e., with or without the face mask; see also Dalmaso et al., 2020b). Therefore, the lack of modulation of gaze cueing as a function of mask condition can be interpreted as a rather straightforward evidence that, at least in some conditions, the gaze cueing effect is not affected by whether or not participants are prevented from seeing the whole face. It should be noted that this reasoning is related to the lack of an overall effect of face masks, although it cannot be excluded that face masks affect gaze cueing in opposite ways in different participants. It would thus be important to explore the eventual role of individual differences as possible intervening factors that either magnify or reduce the attentional response to gaze stimuli embedded in faces wearing a mask.

Interestingly, in a recent study (published after our data collection was ended), Cartaud et al. (2020) explored some aspects regulating social interactions at the time of COVID-19, reporting that the preferred interpersonal distance declared by participants towards a virtual character presented on the screen was smaller when the character wore a face mask rather than when it was presented without a face mask. Furthermore, the declared interpersonal distance was also reduced among individuals who had contracted the virus or who came from areas with a low risk of contagion. Taken together, these results seem to suggest that the willingness to establish a potential social interaction with others at the time of COVID-19 can be influenced by whether they are wearing a face mask or not, and this would be further affected by some individual characteristics that have not been considered here and that could also influence the way we process facial stimuli (Federico et al., 2021). Indeed, in our study, participants were not asked to report if they had contracted COVID-19 nor we collected information about the risk of contagion within the area they were living in at the time of testing, namely two variables that could have also played a role in shaping gaze cueing of attention. This is something future studies could address.

From a purely methodological point of view, social attention abilities have been widely explored by adopting gaze cueing tasks based on manual responses, such as the one employed here. However, in every day social interactions, we tend to explore the surrounding environment around us through eye movements, which can be considered as a more direct and ecological index of visuo-spatial orienting of attention (e.g., Malienko et al., 2018), and can also provide a more fine-grained picture concerning the possible role of social stimuli on social attention (e.g., Dalmaso et al., 2017a, 2017b). In this regard, to the best of our knowledge, only Frank et al. (2021) analysed fixations when participants were presented with faces of individuals either wearing a face mask or not. In particular, they showed that face scanning behaviour was influenced by this protective gear, since the participants spent more time in examining the periorbital region of mask wearers with respect to faces without the face mask. In a similar vein, it will be important to explore whether faces wearing a mask or not can actually impact on gaze-driven attention to a different extent by applying specific oculomotor tasks (e.g., Dalmaso et al., 2020a; Ricciardelli et al., 2002).

One of the main concerns regarding face mask use is related to the impact that such protective gear can have on interpersonal communication (e.g., Mheidly et al., 2020). Indeed, face masks can interfere with identity (e.g., Noyes et al., 2021), and emotion (e.g., Carbon, 2020) recognition. In addition, face masks can hamper the intelligibility of vocal messages (Cohn et al., 2021; Giovanelli et al., 2021). The gaze cueing effect addressed in the present study has been shown to imply the extraction of high-level information about the intentions of the interaction partner and therefore it represents one of the building blocks of social communication (e.g., Colombatto et al., 2020; see also Schmidtmann et al., 2020). The present data indicate that the attentional response to a nonverbal spatial cue conveyed by gaze direction can be observed irrespective of face mask, and hence suggest that this micro-level component of social interaction is preserved. Interestingly, a recent study (Carbon & Serrano, 2021; see also Ruba & Pollak, 2020) has reported that children are not strongly affected by face masks in emotional reading. This, in turn, provides further evidence for the notion that face masks do not necessarily impair face processing mechanisms. To sum up, in two experiments we observed that the tendency to orient our attentional resources towards the spatial location gazed at by others is not affected by whether the face providing the gaze cue is wearing a mask or not, and this emerged among individuals of both a Western and an Eastern country. Even if face masks usage during COVID-19 pandemic is a widely debated topic, and in particular in some contexts (e.g., schools; Spitzer, 2020), we believe that our results, alongside other recent evidence (e.g., Carbon & Serrano, 2021), might be seen as a further incentive to wear face masks in case of airborne diseases (see also Carbon, 2021).

## Appendix A: Questionnaire items

### Section 1: Personal habits

#### A1) In this period of coronavirus-related restrictive measures, on average, how often do you leave your house?

1. Never
2. Less than three times a week
3. Three times a week
4. Four to six times a week
5. Every day

#### B1) In this period of coronavirus-related restrictive measures, when you are away from your house, how often – on average – do you wear face mask when you are outdoors?

1. Never
2. Hardly ever
3. Once in two (50% of the time I am outdoors)
4. Almost always
5. Always

#### C1) In this period of coronavirus-related restrictive measures, when you are away from your house, how often – on average – do you wear face mask when you are indoors?

1. Never
2. Hardly ever
3. Once in two (50% of the time I am indoors)
4. Almost always
5. Always

### Section 2: Concerns about potential infection risk

#### A2) In this period of coronavirus-related restrictive measures, how much important do you consider wearing face mask when you are indoors?

1. Not important at all
2. Not very important
3. I don't know
4. Quite important
5. Extremely important

#### B2) In this period of coronavirus-related restrictive measures, how worried do you feel about a possible coronavirus contagion?

1. Not worried at all
2. Little worried
3. I don't know
4. Quite worried
5. Extremely worried

#### C2) In this period of coronavirus-related restrictive measures, if you are away from your house and you notice a person without a mask coming towards you, how worried do you feel?

1. Not worried at all
2. Little worried
3. I don't know
4. Quite worried
5. Extremely worried
